# Procedural Errors Associated With the Use of Rotary and Reciprocating Endodontic Instruments in Undergraduate Settings: A Scoping Review

**DOI:** 10.1155/ijod/3812332

**Published:** 2026-08-27

**Authors:** Layla Hassouneh, Dalal A. Shamiyah

**Affiliations:** ^1^ Department of Conservative Dentistry, Jordan University of Science and Technology, Irbid, Jordan, just.edu.jo; ^2^ Faculty of Dentistry, Jordan University of Science and Technology, Irbid, Jordan, just.edu.jo

**Keywords:** dental training, procedural errors, root canal treatment, rotary Ni–Ti instruments, undergraduate dental students

## Abstract

**Introduction:**

Root canal treatment (RCT) performed by undergraduate dental students is associated with procedural errors that may compromise outcomes. With the increasing use of rotary nickel–titanium (Ni–Ti) instrumentation, understanding these errors has become important; however, existing evidence remains fragmented. This scoping review aimed to map the available evidence and summarize the reported prevalence of procedural errors associated with RCT using rotary and reciprocating instrumentation in undergraduate dental education.

**Methods:**

This scoping review followed the Arksey and O’Malley framework and PRISMA‐ScR guidelines. A comprehensive search of MEDLINE via PubMed, Scopus, and the Cochrane Central Register of Controlled Trials (CENTRAL) was conducted for studies published between January 1993 and January 2025. Eligible studies included clinical and preclinical investigations involving undergraduate students using rotary or reciprocating Ni–Ti instrumentation and reporting procedural errors. Data were extracted and categorized into preoperative, operative, and post‐operative errors, and prevalence was summarized descriptively.

**Results:**

A total of 878 records were identified, of which 24 studies met the inclusion criteria (14 clinical, 10 preclinical). Operative errors were the most frequently reported, particularly instrumentation‐related errors such as instrument separation, ledge formation, and canal transportation. In clinical studies, instrument separation ranged from 0% to 5.1%, ledge formation from 0% to 29%, canal transportation from 0% to 21%, and perforation from 0% to 31.2%. Obturation‐related errors, particularly underfilling and inadequate filling quality, were also common. In preclinical studies, instrumentation‐related errors such as apical zipping, canal obstruction, and loss of working length were more frequently reported.

**Conclusions:**

Procedural errors remain prevalent in undergraduate endodontic practice despite the use of rotary and reciprocating instrumentation. Instrumentation‐related errors were most consistently reported, while obturation‐related errors were more prominent in clinical studies. The heterogeneity in study design highlights the need for standardized assessment criteria and improved training strategies.

## 1. Introduction

Root canal treatment (RCT) is a technically challenging procedure that requires specialized training and expertise. Therefore, ensuring adequate education and practical training in endodontics is essential as a core learning outcome for undergraduate dental students [1].

In the last four decades, the endodontics field has seen various changes to instruments, materials, and techniques used in the practice. The introduction of rotary nickel–titanium (Ni–Ti) instruments in the late 1980s represents one of the most major advancements in dentistry today [2]. The superior flexibility and efficiency associated with rotary Ni–Ti files have made them a widely utilized tool in today’s endodontic practice as well as frequently used tools in many undergraduate dental programs [3]. Many studies have indicated that rotary instrumentation leads to improved quality and/or consistency of canal preparation, accuracy of shaping of canals, and reduction of overall operative time compared with traditional hand (stainless‐steel) files [4].

Despite these advantages, root canal preparation remains susceptible to procedural errors due to the complex anatomy of the root canal system and the technical sensitivity of the procedure [5]. Procedural errors may occur during different stages of treatment and include canal transportation, ledge formation, apical blockage, perforation, instrument separation, and errors in working length determination such as overfilling or underfilling [6]. These mishaps may compromise the effectiveness of canal debridement and obturation, ultimately affecting treatment outcomes and long‐term tooth prognosis. Previous studies have shown that certain procedural errors could reduce the success rate of endodontic treatment [7]. For example, underfilling and overfilling of the root canal system have been associated with reduced treatment success, while instrument separation may hinder adequate cleaning and shaping of the canal system [8].

Recognizing the importance of preventing such mishaps, undergraduate endodontic education should provide students with the knowledge and practical competence required to minimize procedural errors during RCT. Avoiding complications such as canal transportation, ledge formation, canal blockage, instrument fracture, and perforation are therefore an important component of clinical endodontic training [1]. Accordingly, assisting undergraduate students in demonstrating competence regarding the prevention of such errors is among the primary goals of endodontic education.

To ensure the potential risks are reduced, it is important to realize the nature and frequency of such procedural errors. A number of clinical and preclinical studies have investigated the occurrence of procedural errors during root canal therapy with the use of rotary instruments by undergraduate students [9, 10]. However, since the available data is reported in different studies utilizing different study designs, education facilities, and different types of rotary instruments, there is difficulty in obtaining a full picture of the type and frequency of procedural errors occurring in undergraduate training programs.

Thus, a structured and systematic overview of the available evidence is required to gain insight into how frequent and what types of procedural errors are made by undergraduate students during the use of endodontic rotary instruments. Considering the nature of the available studies, the most effective way to map out the current literature and assess trends, gaps in knowledge, and patterns is through a scoping review. Such synthesis has the potential to identify gaps in knowledge, suggest topics for future research, and provide direction for developing educational approaches and defining best practices relating to endodontic training for undergraduate students.

The research question guiding this review is: *What types of procedural errors have been reported in the literature during root canal treatment using rotary or reciprocating instruments in undergraduate dental education, and what are the reported rates of these errors?*

Accordingly, the aim of this scoping review was to map the available evidence on procedural errors associated with RCT using rotary and reciprocating instrumentation in undergraduate dental education and to summarize the reported prevalence of these errors across clinical and preclinical settings.

## 2. Methodology

A scoping review of the literature was conducted based on the methodology established by Arksey and O’Malley and the PRISMA‐ScR (PRISMA extension for Scoping Reviews) [11, 12].

Focused Question of the review: *What types of procedural errors have been reported in the literature during root canal treatment using rotary or reciprocating instruments in undergraduate dental education*, *and what are the reported rates of these errors?*

The Population–Concept–Context (PCC) framework was used to guide the eligibility criteria for this scoping review.

Population: Permanent teeth undergoing RCT performed by undergraduate dental students in either clinical or pre‐clinical educational settings.

Concept: Procedural errors occurring during RCT using rotary or reciprocating endodontic instrumentation systems.

Context: Undergraduate dental education including both clinical training on patients and preclinical training using extracted teeth or simulated models.

The primary outcomes of interest were procedural errors occurring during RCT, including but not limited to access preparation errors, canal transportation, ledge formation, apical blockage, perforation, instrument separation, and obturation errors. When reported, the frequency or prevalence of these procedural errors was also extracted.

### 2.1. Inclusion and Exclusion Criteria

#### 2.1.1. Inclusion Criteria

For review, studies had to meet the following criteria:

Studies were conducted within undergraduate dental education, including both clinical and preclinical training settings.

Studies investigating RCT using rotary or reciprocating endodontic instrumentation systems, including studies in which these instrumentation were evaluated alone or compared with other instrumentation techniques.

Studies reporting procedural errors occurring during RCT.

Studies conducted on permanent teeth.

Primary research studies, including randomized controlled trials, cohort studies, cross‐sectional studies, retrospective or prospective observational studies, and in vitro preclinical studies.

Articles published in English.

#### 2.1.2. Exclusion Criteria

Studies focusing exclusively on postgraduate students, specialists, or experienced clinicians without relevance to undergraduate training.

Studies investigating instrumentation techniques other than rotary or reciprocating systems (e.g., hand instrumentation only).

Studies in which outcomes related specifically to rotary or reciprocating instrumentation could not be clearly extracted or were not reported separately from other instrumentation techniques.

Case reports, case series, narrative reviews, systematic reviews, scoping reviews, review protocols, editorials, and conference abstracts.

Articles not available in English.

### 2.2. Search Strategy

A comprehensive electronic search of the literature was conducted to identify relevant studies published between January 1993 and January 2025. The final search was conducted on January 21, 2025 in three databases: MEDLINE via PubMed, Scopus, and the Cochrane Central Register of Controlled Trials (CENTRAL). The search strategy was developed using a combination of controlled vocabulary (Medical Subject Headings [MeSH]) and free‐text terms, structured around three main concepts: (1) undergraduate dental education and training, (2) endodontic treatment, and (3) rotary or reciprocating Ni–Ti instrumentation. Boolean operators (AND and OR), truncation, and phrase searching were applied to combine search terms appropriately. The PubMed search strategy was adapted for Scopus and Cochrane CENTRAL by modifying field tags, syntax, and indexing terms as appropriate for each database. The full search strategies for all databases are provided in the Supporting Information. Following the electronic search, a manual search of the reference lists of included articles was performed to identify additional relevant studies that may have been missed during the database search. All retrieved records were imported into EndNote X7 reference management software (Clarivate Analytics, Philadelphia, PA, USA), where duplicate records were identified and removed prior to screening.

### 2.3. Selection of Studies

Identified articles were reviewed for inclusion. An initial examining and screening of the titles and abstracts of all identified articles from electronic searching against both the inclusion and exclusion criteria was conducted. When it was not possible to make a decision based on the title/abstract, the full article was obtained. Only papers that fulfilled all of the eligibility criteria were included. The reason for excluding articles that address the topic of interest but fail to fulfill one or more of the eligibility criteria was recorded. The study selection process was documented using a PRISMA flow diagram in accordance with the PRISMA‐ScR guidelines.

### 2.4. Data Extraction

The data were extracted using a standardized form in Microsoft Office Excel 2016 software (Microsoft Corporation, Redmond, WA, USA). Data of interest were taken from the studies for the composition of a spreadsheet in Excel format, with all included studies containing the following: author(s), year of publication, country, setting (clinical or preclinical), study design, population characteristics (year of study), sample size (number of teeth, canals, or simulated specimens, as applicable), type of teeth, type of NiTi system used, types of procedural errors reported, and prevalence of specific procedural errors. Where possible, results were extracted at the tooth level; however, in preclinical studies using simulated canals, resin blocks, or root‐level analyses, data were recorded at the level reported by the original study. In studies with comparative or mixed instrumentation designs, only data specifically related to the rotary or reciprocating instrumentation groups were extracted and reported, where clearly identifiable.

### 2.5. Data Classification

Due to the heterogeneous nature of studies identified in scoping reviews, including variations in study settings, outcome definitions, and reporting of procedural errors, a standardized classification approach was adopted for the purpose of this review. Procedural errors were categorized into three groups: (i) preoperative errors, (ii) operative errors, and (iii) postoperative errors.

Preoperative errors referred to errors occurring before treatment, such as inaccuracies in diagnosis, treatment planning, or case selection. Operative errors were further subdivided into access‐related, instrumentation‐related, and obturation‐related errors. Access‐related errors included improper access cavity preparation, missed canals, access perforation, and pulpal floor damage. Instrumentation‐related errors included instrument separation, ledge formation, canal transportation, instrumentation‐related perforation, apical zipping, loss of working length, canal obstruction or blocked foramina, elbow formation, stripping, and over‐instrumentation. Obturation‐related errors included under‐extension, over‐extension, and other deficiencies in root filling quality, such as inadequate taper, density, homogeneity, or seal. Postoperative errors included errors occurring after completion of RCT, such as an inadequate coronal seal.

In addition, the reported prevalence of individual procedural errors was extracted and summarized separately for clinical and preclinical studies. Prevalence ranges were calculated only from studies providing extractable numerical data for the specific error. Studies reporting an error without providing a numerical prevalence were recorded but were not included in the calculation of the corresponding range. Only procedural errors associated with rotary or reciprocating instrumentation systems were considered in this review. Data related solely to manual instrumentation techniques were excluded.

## 3. Results

### 3.1. Literature Search and Study Selection

**Figure 1:**
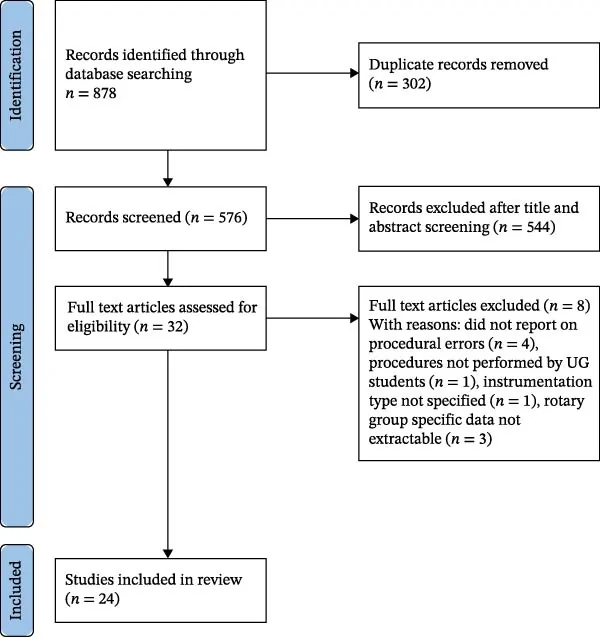
PRISMA flow diagram illustrating the study selection process for the scoping review of procedural errors associated with rotary endodontic instrumentation in undergraduate dental education.

The initial search yielded 878 records. Following duplicate records removal, title, and abstract screening, 32 articles were considered potentially eligible for full‐text assessment. After full‐text review, eight articles were excluded for not meeting the predefined eligibility criteria, resulting in 24 studies included in the final review. The study selection process is summarized in Figure 1, presented according to the PRISMA‐ScR guidelines.

These studies investigated procedural errors and related technical outcomes during RCT performed by undergraduate dental students using rotary or reciprocating Ni–Ti instrumentation systems.

### 3.2. Characteristics of Included Studies

Of the included studies, 10 were conducted in preclinical settings using extracted human teeth, simulated resin blocks, or endodontic training blocks, and 14 were conducted in clinical settings involving patients treated in undergraduate university clinics or hospitals (Table 1). Several studies included comparisons involving rotary or reciprocating Ni–Ti instrumentation and manual or hybrid techniques, while the remaining studies investigated rotary instrumentation systems without a comparator group. The included studies were conducted across a broad range of countries, including Jordan, Romania, the United States, Turkey, Switzerland, Germany, France, China, Saudi Arabia, Taiwan, Brazil, Lithuania, and the United Arab Emirates, demonstrating international interest in the use of rotary instrumentation in undergraduate endodontic education.

**Table 1:** Characteristics of included studies and reported procedural errors during root canal treatment performed by undergraduate dental students.

Study (author, year)	Country	Setting	Study design	Student level	Sample size	Teeth/specimens	NiTi system used	Procedural errors reported (prevalence %)
Preclinical studies								
Namazikhah et al. (2000) [13]	USA	Preclinical	Experimental in vitro	Undergraduate	*n* = 98 teeth (rotary group)	Extracted molar teeth (multiple‐rooted)	0.04‐taper rotary NiTi files (manufacturer not reported)	Loss of working length; ledge formation; apical perforation; perforation; instrument breakage; canal transportation; sealer overfill; gutta‐percha overfill; gutta‐percha underfill; stripping; apical zipping (numerical counts were reported, but percentages were not provided by the authors)
Gluskin et al. (2001) [14]	USA	Preclinical	Experimental in vitro	Undergraduate	*n* = 25 root canals	Mesial roots and canals of extracted mandibular molars (multiple‐rooted teeth)	GT rotary NiTi system (Dentsply Tulsa Dental, Tulsa, OK, USA)	Instrument separation 8% (2/25 rotary canals); canal transportation was assessed quantitatively as canal‐center displacement.
Sonntag et al. (2003) [15]	Germany	Preclinical	Experimental in vitro	Undergraduate	*n* = 450 simulated canals	Simulated single curved canals	FlexMaster rotary NiTi system (VDW GmbH, Munich, Germany)	Apical zipping 17.3%; elbow formation 34.2%; ledge formation 5.3%; under‐extension greater than 2 mm 13.8%; over‐extension beyond the apical foramen 18%; blocked apical foramen 42%; instrument fracture 1.3%.
Steffen et al. (2006) [16]	Germany	Preclinical	Experimental in vitro	Undergraduate	*n* = 36 simulated canals	Simulated single curved canals in resin blocks	ProFile 0.04/0.06 rotary NiTi system (Dentsply Maillefer, Ballaigues, Switzerland)	Instrument failure and canal blockage evaluated (prevalence NR)
Georgelin‐Gurgel et al. (2008) [17]	France	Preclinical	Experimental in vitro	Third‐year undergraduate	*n* = 104 specimens	Extracted human teeth	HeroShaper rotary NiTi system (Micro‐Mega, Besançon, France)	File breakage 7.7%; loss of working length 6.7%; zipping 31.8%; over‐instrumentation 23.1%; blocked foramen 20.2%
Tu et al. (2008) [18]	Taiwan	Preclinical	Experimental in vitro	Fourth‐year undergraduate	*n* = 23 simulated canals	Resin blocks (simulated single curved canals)	ProTaper rotary NiTi system (Dentsply Maillefer, Ballaigues, Switzerland)	Ledge formation 4.35% (1/23 motor‐prepared canals); canal transportation 4.35% (1/23 motor‐prepared canals); instrument separation 0% (0/23 motor‐prepared canals).
Alencar et al. (2010) [19]	Brazil	Preclinical	Experimental in vitro	Fifth‐year undergraduate	*n* = 20 teeth (student group; 60 root canals)	Extracted molar teeth (multiple‐rooted)	ProTaper Universal rotary NiTi system (Dentsply Maillefer, Ballaigues, Switzerland)	Instrument fracture; perforation (coronal, middle, apical); canal transportation (data reported per canal; prevalence not provided separately for student group)
Ünal et al. (2012) [5]	Turkey	Preclinical	Experimental in vitro	Third‐year undergraduate	*n* = 88 root canals	Extracted molar teeth (multiple‐rooted)	ProTaper Universal rotary NiTi system (Dentsply Maillefer, Ballaigues, Switzerland)	Instrument separation 2.27%; loss of working length, and canal straightening (prevalence not reported)
Alrahabi (2015) [20]	Saudi Arabia	Preclinical	Experimental in vitro	Third‐year undergraduate	*n* = 90 canals	Simulated single curved canals in clear resin blocks	ProTaper Universal rotary NiTi system (Dentsply Maillefer, Ballaigues, Switzerland)	Ledge formation 1.1%; instrument separation 5.56%; canal transportation (reported qualitatively; prevalence not reported)
Chirila et al. (2020) [21]	Romania	Preclinical	Experimental in vitro	Undergraduate	*n* = 50 specimens	Extracted single‐rooted teeth, extracted multirooted teeth, and simulated single‐curvature canals in Endo Training blocks	ProTaper Universal rotary NiTi system (Dentsply Maillefer, Ballaigues, Switzerland)	Ledge formation and canal transportation 10.66% (combined); over‐instrumentation 6.66%; apical zipping 3.33%; instrument fracture 4%
Clinical studies								
Kfir et al. (2004) [22]	Israel	Clinical	Prospective controlled clinical trial	Fifth‐year undergraduate	*n* = 69 canals (8‐step NiTi group)	Permanent maxillary and mandibular teeth of all types, including single‐ and multiple‐rooted teeth	Hybrid ProFile–LightSpeed rotary NiTi technique (Dentsply Maillefer; LightSpeed Technology)	Canal transportation 4%; canal obstruction 0%; instrument separation 0%
Cheung and Liu (2009) [23]	China	Clinical	Retrospective observational	Undergraduate + postgraduate	*n* = 110 molars (rotary group)	Permanent molar teeth (multiple‐rooted)	Hybrid technique using ProFile rotary NiTi instruments (Dentsply Maillefer, Ballaigues, Switzerland) and hand instruments	Overall procedural errors 19%; types of errors included ledging, perforation, apical transportation, stripping, and instrument separation
Kelbauskas et al. (2009) [24]	Lithuania	Clinical	Retrospective observational	Undergraduate	*n* = 138 root canals (rotary group only)	Permanent single‐ and multi‐rooted teeth	ProTaper rotary NiTi system (Dentsply Maillefer, Ballaigues, Switzerland)	Underfilling 8.7%; overfilling 5.1%; poor homogeneity/voids 6.7%
Abu‐Tahun et al. (2014) [25]	Jordan	Clinical	Cross‐sectional observational	Fifth‐year undergraduate	*n* = 96 molar teeth	Permanent molar teeth (multiple‐rooted)	ProTaper rotary NiTi system (Dentsply Maillefer, Ballaigues, Switzerland)	Ledge 1.04%; transportation 0%; file separation 1.04%; perforation 0%
Coelho et al. (2017) [26]	USA	Clinical	Retrospective observational	Third‐ and fourth‐year undergraduate	*n* = 1520 treated teeth involving 3194 roots/root canals	Permanent maxillary and mandibular incisors, canines, premolars, and molars, including single‐ and multiple‐rooted teeth	Hybrid techniques using Vortex rotary instruments (Dentsply Tulsa Dental Specialties, Tulsa, OK, USA) and WaveOne reciprocating instruments (Dentsply Maillefer, Ballaigues, Switzerland)	Ledge formation: 1.4% (rotary–hand); 0.5% (reciprocating–rotary); NiTi instrument separation: 0%
Almanei (2018) [27]	Saudi Arabia	Clinical	Pilot study	Undergraduate dental students	*n* = 23 teeth in rotary group	Permanent molar teeth (multiple‐rooted)	ProFile rotary NiTi system (Dentsply Maillefer, Ballaigues, Switzerland), crown‐down; compared with SS hand K‐files	Overall procedural errors: 31.8%; transportation, ledge, perforation, and separated instrument were evaluated but individual prevalence not reported; inadequate length 23.53%; adequate obturation length 65.5%; adequate density 51.28%; adequate taper 47.5%
Hamid et al. (2018) [3]	USA	Clinical	Retrospective observational	Final‐year undergraduate	*n* = 200 molar teeth	Permanent molar teeth (multiple‐rooted)	WaveOne reciprocating system versus a hybrid technique using GT rotary NiTi and hand instruments (Dentsply Tulsa Dental Specialties, Tulsa, OK, USA)	Ledge 8%/29%; instrument separation 1%/4%; canal transportation 13%/21%; perforation 3%/4%; missed canals 2%/1%; inadequate length 29%/52%; inadequate taper 9%/33%; sealer extrusion 50%/52%; inadequate obturation 24% (both groups, derived)
Kurt et al. (2022) [28]	Turkey	Clinical	Retrospective observational	Fifth‐year undergraduate	*n* = 247 teeth (WO group)	Permanent molar teeth (multiple‐rooted)	WaveOne reciprocating NiTi system (Dentsply Maillefer, Ballaigues, Switzerland)	Ledge 1.6%; perforation 0.2%; instrument fracture 5.1%; over‐instrumentation 9.3%; underfilling 13%; overfilling 12.6%; inadequate root canal filling 1.2%
Masoch et al. (2022) [29]	Switzerland	Clinical	Retrospective observational	Fourth‐year undergraduate	*n* = 100 teeth (50 per group; 97 and 81 roots analyzed)	Permanent teeth, including single‐ and multiple‐rooted teeth; individual roots were the unit of analysis	ProTaper Next versus ProFile rotary NiTi systems (Dentsply Maillefer, Ballaigues, Switzerland)	Instrument separation (1 case in ProTaper Next group); no other procedural errors reported (assessment based on radiographic quality score)
Matoug‐Elwerfelli et al. (2022) [30]	Saudi Arabia	Clinical	Retrospective observational	Fourth‐ and fifth‐year undergraduate students	*n* = 109 teeth (rotary group)	Permanent anterior and posterior teeth, including single‐ and multiple‐rooted teeth	ProTaper Universal rotary NiTi system (Dentsply Maillefer, Ballaigues, Switzerland)	Ledge: 2.8%; Zipping: 3.7%; Perforation: 31.2%; Instrument separation: 2.8%; Overall instrumentation mishaps: 38.5%; Obturation‐related mishaps: 37.6%
Tekin et al. (2023) [31]	Turkey	Clinical	Retrospective	UG (4th–5th)	*n* = 280 root canals	Permanent anterior teeth, premolars, and molars, including single‐ and multiple‐rooted teeth	VDW rotary NiTi system (VDW GmbH, Munich, Germany)	Overall procedural errors: 6.4%; Underfilling 5.4%; Overfilling 7.1%; Voids (non‐homogeneous filling) 14.3%; Instrument separation 1.8%; Apical perforation 1.4%; Canal transportation 3.6%; Ledge formation 1.4%
Ameen et al. (2024) [32]	UAE	Clinical	Retrospective cross‐sectional	Fourth‐year undergraduate	*n* = 601 teeth	Permanent anterior and premolar teeth, including single‐ and multiple‐rooted teeth	WaveOne Gold reciprocating NiTi system (Dentsply Sirona, Ballaigues, Switzerland)	Underfilled 2.82%; overfilled 3.66%; inadequate density 3.5%; inadequate taper 1.83%; ledge 0.83%; apical transportation 0%; apical perforation 14.6%; floor damage 0.66%; zipping 0.33%; instrument separation 0.33%
El‐Ma’aita et al. (2024) [10]	Jordan	Clinical	Retrospective observational	Fifth‐year undergraduate	*n* = 104 molars (rotary group)	Permanent molar teeth (multiple‐rooted)	ProTaper Gold rotary NiTi system (Dentsply Sirona, Ballaigues, Switzerland)	Improper access cavity 7.5%; missed canals 5.6%; under‐extension 48.4%; over‐extension 19.2%; improper apical instrumentation size 7.5%; ledge 2.8%; canal transportation 5.2%; access perforation 2.8%; instrumentation perforation 1.4%; strip perforation 0%; separated instrument 3.8%; sealer extrusion 3.8%; insufficient obturation 45.5%; improper coronal seal 35.2%
Medina‐Torres et al. (2024) [33]	USA	Clinical	Retrospective observational	Undergraduate (year NR)	*n* = 368 cases (184 per group)	Permanent anterior and premolar teeth, including single‐ and multiple‐rooted teeth	Vortex Blue rotary system (Dentsply Tulsa Dental Specialties, Tulsa, OK, USA) versus WaveOne Gold reciprocating NiTi system (Dentsply Sirona, Ballaigues, Switzerland)	Overall errors 12.5%; over‐instrumentation 9.8%; transportation 1.6%; ledge 1.1%; instrument separation 0%. WOG: overall errors 24.5%; over‐instrumentation 24.5%; transportation 0%; ledge 0%

The included studies showed considerable methodological heterogeneity (Table 1). Study designs comprised retrospective observational studies, cross‐sectional clinical studies, prospective controlled clinical studies, retrospective in vitro studies, and experimental in vitro investigations. Most preclinical studies evaluated instrumentation performance in molars, curved canals, extracted teeth, or resin blocks, whereas clinical studies assessed the radiographic quality of student‐performed RCTs in molars, premolars, anterior teeth, or mixed tooth groups.

The rotary systems evaluated varied substantially across studies and included ProTaper Universal, ProTaper Gold, ProTaper Next, ProFile, FlexMaster, HeroShaper, Lightspeed, System GT, WaveOne, WaveOne Gold, Vortex Blue, and other hybrid or reciprocating systems.

Across the included studies, the most commonly reported procedural errors were ledge formation, canal transportation, instrument separation, perforation, zipping, loss of working length, over‐instrumentation, underfilling, overfilling, and inadequate obturation‐related outcomes. Considerable variation was observed in both the definitions used and the reported prevalence of these errors across studies. Moreover, some studies reported overall procedural error rates without a detailed breakdown of individual error rates [23].

### 3.3. Types and Prevalence of Procedural Errors

Procedural errors were classified as preoperative, access‐related, instrumentation‐related, obturation‐related, and post‐operative errors. The reported prevalence of these errors varied substantially across the included studies, reflecting differences in the study setting, study design, evaluation methods, tooth type, and unit of analysis (Table 2).

**Table 2:** Classification and reported prevalence ranges of procedural errors, with contributing studies and study counts, in preclinical and clinical undergraduate endodontic settings.

Category	Procedural error	Preclinical studies	Clinical studies
Preoperative	None reported	(*n* = 0)	(*n* = 0)

Access‐related	Improper access cavity	(*n* = 0)	7.5% [10], (*n* = 1)
	Missed canals	(*n* = 0)	1%–5.6% [3, 10], (*n* = 2)
	Access perforation or pulpal floor damage	(*n* = 0)	0.66%–2.8% [10, 32], (*n* = 2)

Instrumentation‐related	Instrument separation, fracture, or failure	0%–8% [5, 14, 15, 17, 18, 20, 21], (*n* = 7)	0%–5.1% [3, 10, 22, 25, 26, 28, 30–33], (*n* = 10)
	Ledge formation	1.1%–5.3% [15, 18, 20], (*n* = 3)	0%–29% [3, 10, 25, 26, 28, 30–33], (*n* = 9)
	Canal transportation	4% [18], (*n* = 1)	0%–21% [3, 10, 22, 25, 31–33], (*n* = 7)
	Instrumentation‐related perforation	(*n* = 0)	0%–31.2% [3, 10, 25, 28, 30–32], (*n* = 7)
	Apical zipping	3.33%–31.8% [15, 17, 21], (*n* = 3)	0.33%–3.7% [30, 32], (*n* = 2)
	Loss of working length	6.7% [17], (*n* = 1)	(*n* = 0)
	Canal obstruction or blocked foramen	20.2%–42% [15, 17], (*n* = 2)	0% [22], (*n* = 1)
	Elbow formation	34.2% [15], (*n* = 1)	(*n* = 0)
	Strip perforation or stripping	(*n* = 0)	0% [10], (*n* = 1)
	Over‐instrumentation or over‐preparation	6.66%–23.1% [17, 21], (*n* = 2)	9.3%–24.5% [28, 33], (*n* = 2)

Obturation‐related	Under‐extension or underfilling	13.8% [15], (*n* = 1)	2.82%–48.4% [10, 24, 28, 31, 32], (*n* = 5)
	Over‐extension or overfilling	18% [15], (*n* = 1)	3.66%–19.2% [10, 24, 28, 31, 32], (*n* = 5)
	Other inadequate obturation outcomes^a^	(*n* = 0)	1.2%–45.5% [3, 10, 24, 27, 28, 30–32], (*n* = 8)

Post‐operative	Improper coronal seal	(*n* = 0)	35.2% [10], (*n* = 1)

*Note:* Only studies reporting extractable numerical data sufficient to determine prevalence were included in the ranges and study counts. Studies reporting an error without sufficient numerical data were excluded from these calculations. Numbers in square brackets indicate the references contributing numerical prevalence data. The letter (*n*) indicates the number of studies included in the reported prevalence range. Studies with multiple rotary or reciprocating groups were counted once.

^a^Includes unspecified inadequate obturation length, inadequate taper, density, homogeneity, voids, and overall inadequate obturation quality.

### 3.4. Preoperative Errors

The reviewed literature demonstrated a clear lack of reporting on preoperative errors. None of the included studies specifically evaluated diagnostic inaccuracies, treatment‐planning errors, or inappropriate case selection.

### 3.5. Access‐Related Errors

Access‐related errors were reported relatively infrequently and were identified only in clinical studies. Improper access cavity preparation was reported in one study [10], while missed canals were reported in two studies [3, 10]. Access‐related perforations and pulpal floor damage were reported in two studies [10, 32]. Overall, access‐related errors were less frequently investigated than instrumentation‐ and obturation‐related outcomes.

### 3.6. Instrumentation‐Related Errors

Instrumentation‐related errors represented the largest and most frequently reported category across both clinical and preclinical studies. The most commonly reported errors were instrument separation, ledge formation, canal transportation, and perforation. Other instrumentation‐related errors included apical zipping, loss of working length, canal obstruction or blocked foramina, elbow formation, stripping, and over‐instrumentation [13, 15–17, 19, 20].

In clinical studies, instrument separation ranged from 0% to 5.1% [3, 26, 28, 31, 32]. Ledge formation ranged from 0.83% to 29% [3, 26, 28, 32], while canal transportation ranged from 0% to 21% [3, 22, 25, 33]. Perforation ranged from 0% to 31.2% [3, 28, 30, 32]. Additional instrumentation‐related errors reported in clinical settings included apical zipping, stripping, and over‐instrumentation, although these outcomes were assessed less consistently.

In preclinical studies, prevalence estimates should be interpreted cautiously because outcomes were frequently assessed at the canal, root, specimen, or simulated‐block level rather than at the tooth level [5, 13–15, 17, 19–21]. Instrument separation was reported in several preclinical studies, with extractable numerical prevalence ranging from 0% to 8% [5, 14, 17, 19, 21], while ledge formation ranged from 1.1% to 5.3% [15, 18, 20]. Canal transportation was reported in several preclinical studies, although only one study provided a separately extractable numerical prevalence of 4% [13, 19, 21]. Perforation was evaluated in some preclinical studies, although no separate extractable numerical prevalence was available for the eligible motor‐driven instrumentation groups [13, 19].

Preclinical investigations more frequently reported detailed instrumentation‐related errors. Apical zipping was reported in several preclinical studies, with extractable prevalence ranging from 3.33% to 31.8% [13, 17], while loss of working length was reported at 6.7% [17]. Canal obstruction or blocked foramina ranged from 20.2% to 42% [15, 17], and over‐instrumentation ranged from 6.66% to 23.1% [17, 21]. Elbow formation and stripping were also reported, although prevalence estimates were not consistently available.

### 3.7. Obturation‐Related Errors

Obturation‐related errors were reported mainly in clinical studies and involved deficiencies in obturation length, taper, density, homogeneity, and overall root‐filling quality [3, 10, 24, 27–29, 32].

Under‐extension or underfilling was reported across clinical studies, with the prevalence ranging from 2.82% to 48.4%, while over‐extension or overfilling ranged from 3.66% to 19.2% [10, 32]. Inadequate root canal filling quality, including deficiencies in taper, density, homogeneity, or seal, was also reported in several studies, with prevalence ranging from 1.2% to 45.5% [3, 10, 24, 28, 29, 32]. These outcomes were more commonly evaluated in clinical studies, where postoperative radiographs were frequently used to assess the technical quality of the treatment.

### 3.8. Postoperative Errors

Postoperative errors were infrequently reported. Improper coronal seal was documented in one clinical study [10]. Incomplete clinical documentation was also identified in some studies as a factor limiting the assessment of certain procedural and post‐treatment outcomes.

### 3.9. Overall Pattern

Overall, instrumentation‐related errors were the most frequently reported procedural errors across both clinical and preclinical settings. Clinical studies more commonly reported major procedural mishaps and obturation‐related deficiencies, whereas preclinical studies identified a broader range of instrumentation‐related errors, including apical zipping, canal obstruction, elbow formation, and loss of working length.

Several studies also explored factors that could influence the occurrence of procedural errors. These included the instrumentation technique used [3, 28, 33], tooth type and anatomical complexity [10, 34], and differences between rotary instrumentation systems [29]. Clinical factors, including the severity of preoperative infection, were also reported to influence treatment outcomes [33]. However, these factors were not consistently investigated across the included studies.

## 4. Discussion

This scoping review mapped the types and reported prevalence of procedural errors associated with rotary or reciprocating instrumentation in undergraduate endodontic education. Across the included studies, instrumentation‐related errors were the most frequently investigated, particularly instrument separation, ledge formation, canal transportation, and perforation. The types of errors reported differed between clinical and preclinical settings, reflecting differences in study design, assessment methods, and units of analysis.

Instrumentation represents a technically demanding stage of RCT for undergraduate students because it requires the integration of tactile perception, spatial awareness, anatomical knowledge, and clinical decision‐making. Errors such as instrument separation, ledge formation, canal transportation, and perforation were consistently documented across both clinical and preclinical investigations [3, 10, 26, 28]. Unlike access cavity preparation and obturation, which generally follow more standardized and closely supervised protocols, canal preparation requires continuous adjustment to the anatomy and response of the canal system. These skills are still developing at the undergraduate level and may contribute to the range of instrumentation‐related errors reported in the included studies.

Interestingly, the type of procedural errors reported differed notably between clinical and preclinical studies, reflecting important methodological differences. Clinical studies, most of which were retrospective and based on radiographic evaluation, tended to report obturation‐related deficiencies and major procedural mishaps [10, 32]. In contrast, preclinical experimental studies, often using extracted teeth or simulated canals, identified a broader range of instrumentation‐related errors, including apical zipping, canal obstruction, elbow formation, and loss of working length [13, 15, 17]. This discrepancy likely reflects the higher sensitivity of laboratory‐based assessment tools compared with conventional radiographic methods used in clinical settings. Moreover, variations in preclinical training, educational exposure, and other potential factors could contribute to differences in procedural outcomes among undergraduate students [35, 36]. As such, the true prevalence of instrumentation‐related errors in clinical practice may be underestimated. Notably, one clinical study reported a substantially higher perforation rate (31.2%), highlighting the variability in reported outcomes and potential influence of case selection and assessment criteria in clinical studies [30].

The methods used to assess RCT also differed substantially between preclinical and clinical studies. Preclinical investigations commonly used extracted teeth or simulated canals under standardized conditions and employed more detailed assessment methods, including direct inspection and advanced imaging techniques, which facilitated the detection of subtle changes in canal anatomy [14, 19]. These methods enabled the detection of subtle instrumentation‐related changes, such as canal transportation, straightening, apical zipping, blockage, and loss of working length [5, 14, 15, 19]. In contrast, most clinical studies relied on a retrospective review of patient records and two‐dimensional periapical radiographs. These assessments were more suitable for identifying obturation length, density, homogeneity, instrument separation, and obvious perforations but were less sensitive for detecting three‐dimensional changes in canal anatomy or minor transportation [3, 24, 28, 32].

An important observation in the current review is that there were large differences in the study designs and sample sizes of the included studies. Most preclinical studies have used small sample sizes (ranging from a few dozen to a few 100 canals), whereas most clinical studies used larger sample sizes that included hundreds or thousands of teeth [26]. Furthermore, most of the included clinical studies were designed as retrospective observational studies, whereas most of the preclinical studies were designed as experimental studies. This degree of variability is characteristic of scoping reviews and supports the use of such methodology to ensure the inclusion of all relevant evidence [12]. However, the degree of variability limits the ability to compare studies directly and complicates the ability to generalize the results from the studies.

The transition from hand instrumentation to rotary systems in undergraduate training was evident in many of the included studies, with several directly comparing these techniques. While rotary instrumentation offers advantages such as improved efficiency and more standardized canal shaping, it also introduces new challenges for novice operators. The different instrumentation systems evaluated may have contributed to the variation in the procedural errors reported across studies. Instrument systems differ in alloy treatment, cross‐sectional design, taper, flexibility, number of instruments, and kinematics, which can influence canal‐centering ability, preservation of the original canal anatomy, and resistance to cyclic or torsional failure [37–39]. Simplified single‐file or reciprocating protocols may be easier for novice operators to follow; however, their performance remains dependent on adequate access, glide path preparation, working length control, and adherence to the recommended technique. Comparisons should therefore be interpreted cautiously because the included studies differed in tooth anatomy, operator experience, training protocols, and assessment methods, preventing procedural errors from being attributed solely to the instrument system used [3, 29, 33].

ProTaper‐based systems were among the most frequently evaluated systems in the included studies and were associated with instrumentation‐related errors such as canal straightening, ledge formation, and transportation, particularly in curved canals [5, 10]. These findings may reflect differences in file design, taper, canal anatomy, and the adequacy of glide path preparation. Some studies comparing rotary and reciprocating systems reported fewer selected errors with reciprocation, including working‐length loss and underfilling; however, these findings were not consistent across studies and may also have been influenced by differences in case complexity, operator experience, and training protocols [3, 40].

The tooth type also played a significant role in procedural error occurrence. Molar teeth were consistently associated with higher rates of errors, likely due to their complex anatomy, limited accessibility, and the presence of multiple canals [3, 5, 10]. Errors such as missed canals, ledge formation, and obturation deficiencies were more frequently reported in molars compared with anterior or premolar teeth, which aligns with previous reports highlighting the increased technical difficulty of posterior teeth [41]. These findings reinforce the importance of careful case selection in undergraduate training and support a gradual progression from simpler to more complex cases.

The findings of this review should be viewed as a reflection of the unique characteristics of undergraduate clinical education. Undergraduate students have less development of basic clinical skills than experienced clinicians and will continue to develop hand–eye coordination and procedural planning throughout their training. Therefore, the factors influencing their performance include limited clinical experiences, variability in clinical supervision, and time constraints imposed by academic settings. Accordingly, the types of procedural errors that will be observed among undergraduate students will be quite different from the types of procedural errors observed among general dentists or specialists. In fact, previous research has demonstrated that more than half of undergraduate students will report at least one procedural error during their education [9]. Therefore, the data obtained from general dentists or specialists may not accurately reflect the challenges faced by undergraduate dental students [41]. This review focuses on undergraduate students and provides information that is particularly relevant to dental education.

The review highlights several important gaps in the existing literature. Preoperative errors, such as diagnostic or case selection errors, were rarely reported. Similarly, postoperative errors, particularly those related to coronal seal and long‐term treatment outcomes, were underrepresented. Only a limited number of studies addressed such aspects [10]. This may be partly due to the reliance on retrospective radiographic data as well as inconsistencies in documentation across studies. Furthermore, differences in assessment methods and units of analysis (tooth vs. canal level) could also complicate the interpretation of prevalence data.

The methodological heterogeneity across the included studies in the current review is considered a key limitation. Variability in study design, evaluation methodology, tooth type, and outcome measures made synthesis of the results quantitatively difficult. Very few clinical studies accounted for potential confounding factors such as student experience level, level of supervision, and case difficulty, which could have an impact on the prevalence of procedural errors but have not been adequately evaluated in the studies.

The findings of this review have practical implications for undergraduate endodontic education. Curricula should explicitly link commonly reported instrumentation‐related errors—particularly instrument separation, ledge formation, canal transportation, perforation, and loss of working length—to their causes, prevention, recognition, and management. These outcomes could be incorporated into competency‐based learning objectives, standardized operating protocols, case‐based teaching, and formative assessment [1].

Preclinical training should introduce rotary and reciprocating instrumentation gradually, beginning with simple simulated canals and progressing to curved canals and anatomically complex extracted teeth. Repeated practice should emphasize access preparation, glide path establishment, working length control, maintenance of canal anatomy, and adherence to the recommended file sequence. More frequent technical evaluation and targeted mentor feedback after practical exercises may help students identify recurrent errors and improve performance before entering clinical training [35].

Before independently performing clinical endodontic procedures, students should demonstrate minimum competencies in safe access preparation, accurate working‐length determination, reproducible glide path preparation, controlled canal instrumentation, recognition of procedural errors, and acceptable obturation quality. In clinical training, case difficulty should be matched to demonstrated competence, with standardized checklists and closer supervision during high‐risk stages. Students who repeatedly demonstrate technical errors should receive additional simulation‐based training and individualized feedback before progressing to more complex cases [10].

## 5. Conclusion

In conclusion, this scoping review showed that instrumentation‐related errors were the most frequently reported procedural errors in undergraduate RCT using rotary or reciprocating systems. These mainly included instrument separation, ledge formation, and canal transportation. Obturation‐related errors were also common in clinical studies, particularly underfilling. Errors were more prevalent in molar teeth, reflecting their greater anatomical complexity. Variability in study design, assessment criteria, and institutional settings influenced the findings, emphasizing the need for standardized evaluation methods and targeted educational interventions to improve endodontic training outcomes. Such data would support evidence‐based improvements in endodontic education and ultimately contribute to better patient care.

## Author Contributions

**Layla Hassouneh:** conceptualization, methodology, formal analysis, data curation, writing – original draft, writing – review and editing, supervision, project administration. **Dalal A. Shamiyah:** investigation, data curation, writing – review and editing.

## Funding

This study did not receive any specific funding.

## Disclosure

All authors have read and approved the final version of the manuscript. The corresponding author, Layla Hassouneh, had full access to all of the data in this study and takes complete responsibility for the integrity of the data and the accuracy of the data analysis.

## Ethics Statement

Ethical approval was not required because this scoping review used only previously published data and involved no human participants or identifiable information.

## Conflicts of Interest

The authors declare no conflicts of interest.

## Supporting Information

Additional supporting information can be found online in the Supporting Information section.

## Supporting information


**Supporting Information** Additional supporting information related to this scoping review is available as a Supporting file. Supporting Information: Detailed search strategies for all databases used in this review, including PubMed, Scopus, and Cochrane Library. The complete electronic search strategy, including keywords, MeSH terms, Boolean operators, and search combinations, is provided to ensure transparency and reproducibility of the search process.

## Data Availability

The authors confirm that the data supporting the findings of this study are available within the article and its Supporting Information.
