# Supplementary material for: Procedural Errors Associated With the Use of Rotary and Reciprocating Endodontic Instruments in Undergraduate Settings: A Scoping Review
Source: Int J Dent. 2026 Aug 27;2026:3812332. doi: 10.1155/ijod/3812332 (PMC13519951; doi:10.1155/ijod/3812332)
Supplement: Supplementary file 1 [file IJOD-2026-3812332-s001.docx]

# Supplementary Material 1: Detailed Search Strategies for All Databases

Supplementary Table 1: Complete electronic search strategies used for each database

| **Database** | **Search strategy** | **Filters/limits** | **Date searched** |
| --- | --- | --- | --- |
| MEDLINE via PubMed | #1: "students, dental"[MeSH Terms] OR "dental student*"[Title/Abstract] OR "undergraduate dental student*"[Title/Abstract] OR "dental undergradu*"[Title/Abstract] OR "student*"[Title/Abstract] OR "undergraduate*"[Title/Abstract] OR "dental education"[Title/Abstract] OR "education"[Title/Abstract] OR "training"[Title/Abstract] OR "preclinical"[Title/Abstract] OR "clinical training"[Title/Abstract] OR "novice operator*"[Title/Abstract] OR "inexperienced operator*"[Title/Abstract] #2: "Endodontics"[MeSH Terms] OR "Root Canal Therapy"[MeSH Terms] OR "endodontic"[Title/Abstract] OR "Endodontics"[Title/Abstract] OR "root canal treatment"[Title/Abstract] OR "Root Canal Therapy"[Title/Abstract] OR "root canal preparation"[Title/Abstract] OR "canal preparation"[Title/Abstract] #3: "rotary"[Title/Abstract] OR "rotary instrument*"[Title/Abstract] OR "rotary instrumentation"[Title/Abstract] OR "reciprocating"[Title/Abstract] OR "reciprocating instrument*"[Title/Abstract] OR "reciprocating file*"[Title/Abstract] OR "reciprocating system*"[Title/Abstract] OR "engine-driven"[Title/Abstract] OR "engine-driven"[Title/Abstract] OR "nickel-titanium"[Title/Abstract] OR "nickel-titanium"[Title/Abstract] OR "NiTi"[Title/Abstract] OR "endodontic file*"[Title/Abstract] OR "niti file*"[Title/Abstract] OR "rotary file*"[Title/Abstract] #1 AND #2 AND #3 | English; January 1993–January 2025 | 21-Jan-25 |
| Scopus | ( TITLE-ABS-KEY ( "dental student*" OR "undergraduate dental student*" OR "dental undergradu*" OR student* OR undergraduate* OR "dental education" OR education OR training OR preclinical OR "clinical training" OR "novice operator*" OR "inexperienced operator*" ) ) AND ( TITLE-ABS-KEY ( endodontic OR endodontics OR "root canal treatment" OR "root canal therapy" OR "root canal preparation" OR "canal preparation" OR "root canal" ) ) AND ( TITLE-ABS-KEY ( rotary OR "rotary instrument*" OR "rotary instrumentation" OR reciprocating OR "reciprocating instrument*" OR "reciprocating file*" OR "reciprocating system*" OR "engine driven" OR "engine-driven" OR "nickel titanium" OR "nickel-titanium" OR NiTi OR "endodontic file*" OR "NiTi file*" OR "rotary file*" ) ) | English; January 1993–January 2025 | 21-Jan-25 |
| Cochrane CENTRAL | (dental student* OR undergraduate* OR dental education OR training OR preclinical OR "clinical training" OR "novice operator*" OR "inexperienced operator*") AND (endodontic OR "root canal" OR "root canal treatment" OR "root canal therapy" OR "canal preparation") AND (rotary OR "rotary instrument*" OR "rotary instrumentation" OR reciprocating OR "reciprocating instrument*" OR "reciprocating file*" OR "reciprocating system*" OR "engine driven" OR "engine-driven" OR "nickel titanium" OR "nickel-titanium" OR NiTi OR "endodontic file*" OR "rotary file*") in Title Abstract Keyword - (Word variations have been searched) | January 1993–January 2025 | 21-Jan-25 |
